# Clear Cell Sarcoma-Like Tumor of the Gastrointestinal Tract, Presenting as a Second Malignancy after Childhood Hepatoblastoma

**DOI:** 10.1155/2014/984369

**Published:** 2014-02-17

**Authors:** Khin Thway, Ian Judson, Cyril Fisher

**Affiliations:** ^1^Sarcoma Unit, Royal Marsden Hospital, London SW3 6JJ, UK; ^2^Department of Histopathology, The Royal Marsden NHS Foundation Trust, 203 Fulham Road, London SW3 6JJ, UK

## Abstract

Clear cell sarcoma-like tumor of the gastrointestinal tract (CCSLGT) is a rare malignant neoplasm arising within the wall of the small bowel, stomach, or large bowel, predominantly in children and young adults. It is an aggressive tumor with a high rate of local recurrence, metastases, and early death from disease. Histologically, it is composed of relatively monomorphic ovoid or round cells with clear to eosinophilic cytoplasm, arranged in sheets and sometimes papillary or alveolar architectures, often with CD68-positive osteoclast-like giant cells in variable numbers, and is associated with *EWSR1-CREB1* gene fusions. Its pathogenesis is unknown, and histologically it can be easily confused with a variety of intra-abdominal neoplasms. We describe a case of CCSLGT with molecular characterization, presenting as an acutely obstructing small bowel mass in a 33-year-old male, which occurred as a second malignant neoplasm 20 years after treatment with surgery, radiotherapy, and cisplatin and doxorubicin chemotherapy for childhood hepatoblastoma. This gives further insight into the clinical setting of this highly aggressive neoplasm and highlights the use of radiation therapy as a possible etiologic factor.

## 1. Introduction

Clear cell sarcoma-like tumor of the gastrointestinal tract (CCSLGT) is a rare malignant neoplasm arising within the wall of the small bowel, stomach, or large bowel, predominantly in children and young adults. It is associated with high rates of local recurrence, metastasis, and early deaths from disease. Histologically it consists of relatively monomorphic ovoid or epithelioid cells with clear to eosinophilic cytoplasm arranged in sheets and papillary or alveolar architectures, with variable numbers of CD68-positive osteoclast-like giant cells. Because of the nonspecific immunoprofile of focal S100 protein expression and general lack of immunoreactivity to other antibodies, it may be misdiagnosed as a variety of neoplasms, including melanoma, malignant peripheral nerve sheath tumor, or gastrointestinal stromal tumor (GIST). We describe a case arising in the small bowel wall and harboring *EWSR1-CREB1* gene fusion by reverse transcription polymerase chain reaction, which occurred in a 33-year-old male who had previously had surgery, irradiation, and chemotherapy for childhood hepatoblastoma. This expands the clinical spectrum of this highly aggressive neoplasm and highlights the possibility of radiation therapy as a predisposing cause.

## 2. Case History

A 33-year-old Caucasian male presented to the Emergency Department of his local hospital with worsening abdominal pain, exacerbated by eating, which he had had intermittently for the preceding five months and which had led to weight loss of two stone over the last three months. On clinical examination, the patient had abdominal distension and pain in the lower right quadrant, along with high pitched bowel sounds. 20 years previously, at the age of 13, he had been treated for hepatoblastoma with six cycles of PLADO regimen (cisplatin and doxorubicin) chemotherapy followed by partial hepatectomy and radiotherapy in the context of a Society of Pediatric Oncology Liver Cancer (SIOPEL) Group clinical trial. He had been fit and well since, without detectable late effects from this regimen. The patient's father and grandmother had both been previously successfully treated for colon cancer in their 60s, but there was no other family history of cancer. Abdominal X-ray showed prominent small bowel loops and no air in the rectum. Abdominal computed tomography (CT) scan revealed distal small bowel obstruction due to an intussusception in the right lower quadrant of the abdomen. A 3 cm mass was present at the apex of the intussusception, along with two enlarged mesenteric lymph nodes. The imaging also showed features relating to the patient's previous treatment, with extensive metallic artefact deep into the liver, abutting a patent portal vein with an unusual course of marked anterior looping, a very small pancreatic head and neck, and absent gallbladder, with clips around the portal vein at the head of the pancreas. No focal abnormalities were seen within the liver or spleen. The small bowel mass was resected with clear margins and a primary anastomosis formed. The patient made a good recovery and was discharged shortly afterwards, and owing to the local hospital diagnosis of gastrointestinal stromal tumor (GIST) he was referred to our tertiary center. The diagnosis was revised to clear cell sarcoma-like tumor of gastrointestinal tract, as below. Given the absence of residual disease and the largely resistant nature of this neoplasm to standard chemotherapeutic regimens, there was no role for adjuvant chemotherapy, and hence the treatment plan was for close clinical and radiological followup. Two months later, the patient was admitted to his local hospital with a two-week history of fevers with night sweats and rigors. No specific source of infection was noted clinically, but ultrasound scan of the abdomen showed abnormal liver lesions which were thought to represent possible abscesses. Treatment with intravenous metronidazole and tazocin was commenced for pyrexia of unknown origin and he was again referred to our institution. CT scan showed rapid progression of locally recurrent disease with the development of peritoneal nodules, widespread liver metastases, and prominent mesenteric adenopathy. The liver was biopsied and showed metastatic tumor with similar morphology and immunoprofile to that in the small bowel. Owing to the rapid disease progression, the patient did not wish to be considered for systemic therapy and died from advanced malignancy seven months after initial presentation with this neoplasm.

## 3. Materials and Methods

The histopathological features were noted, and a comprehensive immunohistochemical panel was applied (antibody dilutions and sources are in Table 1). Molecular genetic and molecular cytogenetic analyses were performed on formalin fixed, paraffin embedded (FFPE) material for each case, for *EWSR1 *rearrangements by fluorescence *in situ* hybridization (FISH), and for specific fusion transcripts by reverse transcription polymerase chain reaction (RT-PCR). For FISH, 1 *μ*m thick FFPE sections were dewaxed overnight at 60°C, treated with hot buffer wash at 80°C (2-3 hr) and then proteolytic enzyme treatment at 37C°, and washed in distilled water and then an alcohol series before addition of *EWSR1 *DNA probes (Abbott Laboratories Ltd., UK). Hybridization was performed overnight according to manufacturer's protocols. RT-PCR was performed to assess* EWSR1-CREB1* and* EWSR1-ATF *fusion transcripts, according to standard or previously described methods [1, 2].

## 4. Results (Histopathological, Immunohistochemical, and Molecular Genetic and Cytogenetic Findings)

### 4.1. Primary Neoplasm

**Figure 1:**
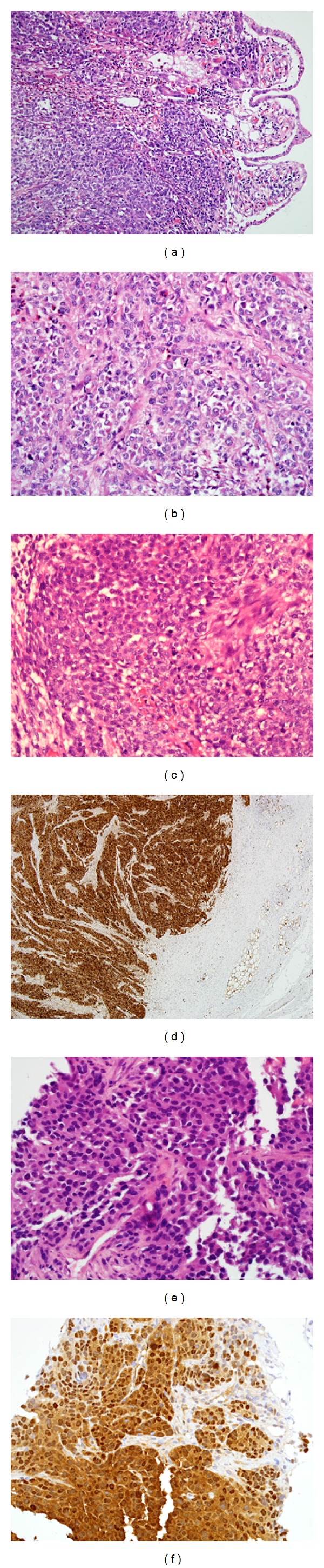
(a) Clear cell sarcoma-like tumor of the gastrointestinal tract (CCSLGT). Low power view shows diffuse infiltration of the wall of the small bowel completely destroying the muscularis propria and with obliteration of the intestinal mucosa leaving just a thin strip of epithelium overlying each of the villi. (b) The tumor comprises sheets and vague nests of epithelioid or ovoid cells which show moderate nuclear atypia and amphophilic or clear cytoplasm. The nests are much more ill defined than those seen in conventional soft tissue clear cell sarcoma of tendons and aponeuroses. (c) At higher power, the cells show medium sized ovoid to occasionally spindled vesicular nuclei and often small nucleoli. There are prominent mitotic figures. Here, the cells are present in a diffuse sheet-like pattern without discernible nested architecture. (d) There is diffuse nuclear and cytoplasmic expression of S100 protein. S100 protein expression without staining for melanocytic markers is typical of CCSLGT, although some cases may show patchier S100 protein immunoreactivity. Note the diffuse infiltration of the small bowel muscularis propria and tumor proximity to serosal fat. (e) Metastatic CCSLGT in the patient's subsequent liver biopsy. The metastatic tumor has similar morphology, although it contains cells with amphophilic cytoplasm and no clear cytoplasm or any nested pattern. The lack of characteristic cytologic or immunophenotypic features and the lack of familiarity of pathologists with this neoplasm can understandably lead to confusion with other metastatic tumors with epithelioid or ovoid cell morphology. (f) Diffuse S100 protein expression is also present in the liver metastasis.

Grossly, the specimen consisted of two segments of small intestine, the larger approximately 30 cm in length with attached omentum. 14 cm from one margin there was a polypoid 4 cm diameter tumor obstructing the lumen. The serosa was unremarkable, as was the smaller piece of bowel. Histologically, the mass was an ulcerated polypoid infiltrative hypercellular neoplasm involving the full thickness of the small bowel wall (Figure 1(a)) and extending into the adjacent adipose tissue. The tumor was predominantly composed of irregular nests of fairly uniform epithelioid and short spindle cells with ovoid vesicular nuclei, inconspicuous nucleoli, and moderate amounts of amphophilic to focally clear cytoplasm (Figures 1(b) and 1(c)). There were areas of sheet-like and fascicular architecture (Figure 1(b)) and focal cellular dyscohesion imparting an alveolar-like pattern. In areas, cells were enlarged with increased nuclear pleomorphism, irregular nuclear outlines, and prominent nucleoli, and there were small numbers of multinucleated tumor cells. The mitotic index exceeded 20/10 hpf in these foci and included atypical forms. Focal necrosis was present as well as a patchy chronic inflammatory infiltrate. No osteoclast-like giant cells, melanin pigment, or packeted or short fascicular architecture typical of conventional clear cell sarcoma (of tendons and aponeuroses) (CCS) was present.

The tumor was strongly and diffusely positive for S100 protein (Figure 1(d)), with focal weak staining for epithelial membrane antigen (EMA), CD56, and neurone-specific enolase (NSE). It was negative for all other markers: CD117, DOG1, desmin, SMA, h-caldesmon, HMB45, MelanA, CD34, AE1/AE3, Cam5.2, chromogranin, synaptophysin, neurofilament, HCG, and AFP. INI1 was retained in nuclei. There was prominent lymphovascular permeation, and metastatic tumor was present in three of eleven regional lymph nodes. *EWSR1-CREB1* fusion transcript was detected by real-time RT-PCR and confirmed by direct sequencing analysis. *EWSR1-ATF1 *fusion transcripts were undetectable by RT-PCR. FISH using break-apart probes for *EWSR1* at 22q12 showed cells with a split *EWSR1 *signal, indicating the presence of a translocation involving the *EWSR1* gene at 22q12. The findings were consistent with clear cell sarcoma-like tumor of the gastrointestinal tract.

### 4.2. Metastatic Neoplasm

The core biopsies from the liver showed moderately congested and chronically inflamed liver parenchyma containing cellular tumor comprising sheets and vague nests of uniform epithelioid cells with hyperchromatic ovoid nuclei, moderate amounts of amphophilic cytoplasm (Figure 1(e)), and prominent mitoses. There was strong and diffuse S100 protein expression (Figure 1(f)), and the features were similar to those in the small bowel neoplasm and consistent with metastatic clear cell sarcoma-like tumor of the gastrointestinal tract.

## 5. Discussion

We describe a case of clear cell sarcoma-like tumor of the gastrointestinal tract, arising in the small bowel of an adult male, which harbored *EWSR1-CREB1* gene fusion and occurred 20 years after chemotherapy, resection, and radiation treatment for childhood hepatoblastoma. CCSLGT is a rare and recently characterized neoplasm, which typically behaves in an aggressive manner with a high rate of local recurrence, metastases (often at presentation), and early death from disease [3–6], in contrast to the longer survival of patients with conventional (soft tissue) CCS. CCSLGT occurs predominantly in the small or large bowel and stomach of children and young adults [4–6], growing as a transmural, often ulcerating mass. Its etiology is unknown, and as yet there is no consensus on the use or benefit of adjuvant or targeted therapies. *EWSR1-CREB1* activates the melanocyte transcription factor MITF, which in turn activates transcription of c-Met, an oncogenic receptor tyrosine kinase recently shown to be activated in clear cell sarcoma [7]. Inhibitors of MET are currently being studied in early clinical trials [8, 9].

CCSLGT was first described by Zambrano et al. in 2003 [4, 10] and is characteristically composed of fairly monomorphic ovoid or round cells with clear to eosinophilic cytoplasm and small or inapparent nucleoli, arranged in sheets or papillary or alveolar architectures [4, 6]. Spindling of cells and macronucleoli are rare, and the well-formed nests typical of conventional CCS are absent. Large epithelioid cells [11] and pleomorphism are occasionally described [12] and there is usually prominent mitotic activity and focal necrosis. CD68-positive osteoclast-like giant cells are often interspersed in variable distribution and numbers [4], but the tumoral giant cells of conventional CCS are not seen. CCSLGT is at least focally positive for S100 protein but does not express melanocytic markers such as HMB45 or MelanA. Tumors can also express neuroendocrine markers focally, such as chromogranin, synaptophysin, NSE, or CD56 [3, 5, 6, 13–17]. Based on this and ultrastructural findings showing lack of evidence of melanocytic differentiation but sometimes features of neural differentiation [3, 4, 6], it was proposed that CCSLGT might be redesignated as gastrointestinal neuroectodermal tumor (GNET) [17]. Most of the small numbers of CCSLGT reported have shown *EWSR1-CREB1* fusions, although some harbor *EWSR1-ATF1 *fusions that are associated with conventional CCS. While the latter can also arise in the gastrointestinal tract, most contain *EWSR1-ATF1* fusions [3, 18–22]. The clinical behavior of gastrointestinal clear cell sarcomas is noted to be aggressive, regardless of the fusion type [6].

This study describes a molecularly confirmed CCSLGT, with *EWSR1-CREB1* fusion transcript that occurred as a rapid and fatal second malignancy in a patient who had had chemotherapy and radiation treatment for childhood hepatoblastoma two decades previously. The treatment of hepatoblastoma has been one of the success stories of pediatric oncology over the last few decades, with a dramatic increase in overall survival rates that are now as high as 70%. This has been in no small part due to the aggressive treatment regimens administered to young patients. However survivors of childhood cancer are at known risk for developing second malignant neoplasms, and CCSLGT has been rarely documented as a subsequent malignancy in this group of patients. One case of CCSLGT has been reported as a second malignancy following neuroblastoma in infancy [23]. Another patient developed CCSLGT nearly 15 years after treatment with very low dose therapeutic radiation (4.5 Gy) for stage 4S neuroblastoma [24]. While two reports describe clear cell sarcoma presenting as a second malignant neoplasm following prior therapy for acute leukemia [4, 13], one appears histologically to be more in keeping with conventional CCS [13], while the other is reported as harboring a t(12;22) translocation in keeping with *EWSR1-ATF1* rearrangement [4]. While we were unable to obtain documentation of the radiation fields for the patient's hepatoblastoma, the site of the CCSLGT is likely to have received some radiation and it is commonly seen that secondary sarcomas occur at the periphery of the radiation field. Radiotherapy delivery was much less precise in the era when the patient was treated, increasing the likelihood that this was a significant factor. There are isolated case reports of secondary cancers, both leukemia and sarcoma, following treatment with cisplatin and doxorubicin, principally given for the treatment of osteosarcoma, but such cases appear to be rare.

Given the rarity of this neoplasm and the fact that it can easily mimic more common lesions occurring at intra-abdominal sites including conventional CCS, metastatic melanoma, GIST, malignant perivascular epithelioid cell neoplasm (PEComa), and clear cell carcinomas such as those from the kidney, it seems likely that the true incidence of CCSLGT is underrepresented because it has been misdiagnosed due to unfamiliarity of surgical pathologists with the entity and the largely nonspecific immunophenotype.

In summary, we report a case of CCSLGT, which occurred as a second malignant neoplasm in a 33-year-old male who had had previous chemotherapy and irradiation for childhood hepatoblastoma. The etiology of these rare and largely rapidly fatal neoplasms remains unknown, and this report draws further attention to childhood irradiation as a possible precipitating factor. It remains to be seen whether CCSLGT will emerge as a potential late complication of the presumed irradiation component of the treatment for hepatoblastoma or other pediatric neoplasms or whether this case simply represents an unrelated second malignancy in a patient who had increased susceptibility to neoplasia compared to the general population. Documentation of any CCSLGT is nevertheless important due to its extreme rarity, particularly when occurring in patients with relevant past medical histories, so that we can come closer to establishing its cause. While effective treatments are not yet available for this highly aggressive neoplasm, future targeted therapies that inhibit the function of the *EWSR1-CREB1* fusion oncogene or its relevant downstream pathways may be effective at treating disease in the future.

## Figures and Tables

**Table 1:** Antibodies used for immunohistochemistry.

Antibody	Source	Dilution
AE1/AE3	Zymed Laboratories, CA, USA.	1 : 50
MNF116	Dako, Glostrup, Denmark.	1 : 400
Cam5.2	Becton Dickinson, Plymouth, UK.	1 : 10
EMA	Dako, Glostrup, Denmark.	1 : 400
Desmin	Dako, Glostrup, Denmark.	1 : 50
SMA	Dako, Glostrup, Denmark.	1 : 200
S100 protein	Dako, Glostrup, Denmark.	1 : 1500
HMB45	Dako, Glostrup, Denmark.	1 : 100
MelanA	Dako, Glostrup, Denmark.	1 : 25
CD34	Novocastra Laboratories, Newcastle upon Tyne, UK.	1 : 30
CD45	Dako, Glostrup, Denmark.	1 : 20
CD56	Invitrogen, Camarillo, CA, USA.	1 : 50
CD117	Dako, Glostrup, Denmark.	1 : 500
DOG1	Novocastra Laboratories, Newcastle upon Tyne, UK.	1 : 50
Chromogranin	Dako, Glostrup, Denmark.	1 : 300
GFAP	Dako, Glostrup, Denmark.	1 : 200
Neurofilament	Dako, Glostrup, Denmark.	1 : 200
NSE	Dako, Glostrup, Denmark.	1 : 600
Synaptophysin	Dako, Glostrup, Denmark.	1 : 100
INI1	Becton Dickinson, Plymouth, UK.	1 : 100
MIB1	Dako, Glostrup, Denmark.	1 : 100
